# The prevalence and antifungal susceptibility profile of *Candida dubliniensis* and *Candida albicans* in the respiratory tract of patients with cystic fibrosis

**DOI:** 10.1128/spectrum.01907-25

**Published:** 2025-10-27

**Authors:** Emily Krantz, Bodil Jönsson, Marita Gilljam, Anders Lindblad, Bahman Abedzadeh, Roger Karlsson, Nahid Kondori

**Affiliations:** 1 Department of Internal Medicine and Clinical Nutrition, Institution of Medicine, Sahlgrenska Academy, University of Gothenburg 70712, Gothenburg, Sweden; 2 Gothenburg CF-Centre, Department of Respiratory Medicine, Sahlgrenska University Hospital 56749, Gothenburg, Sweden; 3 Department of Infectious Diseases, Institute of Biomedicine, Sahlgrenska Academy, University of Gothenburg 70712, Gothenburg, Sweden; 4 Department of Clinical Microbiology, Region Västra Götaland, Sahlgrenska University Hospital 56749, Gothenburg, Sweden; 5 Gothenburg CF-Centre, Department of Pediatrics, Queen Silvia Children’s Hospital, Gothenburg, Sweden; University of Maryland School of Medicine, Baltimore, Maryland, USA

**Keywords:** cystic fibrosis, *Candida dubliniensis*, *Candida albicans*, antifungal susceptibility, fungal colonization

## Abstract

Respiratory infections are the leading cause of mortality and morbidity among cystic fibrosis (CF) patients. Pathogenic bacteria and fungi such as *Pseudomonas aeruginosa*, *Staphylococcus aureus*, and various yeasts as well as filamentous fungi are commonly identified opportunistic microorganisms recovered in the sputa from CF patients. The clinical significance of yeasts, particularly *Candida* species, in patients with CF is not fully understood. Furthermore, the emergence of antifungal resistance in clinical settings is a global concern. This study aimed to determine the prevalence of *Candida* species with special focus on *Candida dubliniensis* and *Candida albicans* in sputum samples from patients with CF and evaluate their susceptibility to antifungal agents. A total of 968 culture-positive sputum samples were collected from 193 patients with CF over a period of 1 year. Fungal isolates were identified to the species level using matrix-assisted laser desorption ionization-time-of-flight mass spectrometry. The antifungal susceptibility of *Candida* isolates was determined using Sensititre YeastOne panels. The most commonly isolated yeast species in these sputum samples were *C. dubliniensis* (13%) and *C. albicans* (32%). Colonization with *C. dubliniensis* or *C. albicans* was not associated with a deteriorated lung function. Voriconazole had the lowest minimum inhibitory concentration (MIC) against *C. dubliniensis* and *C. albicans*. Itraconazole showed higher MIC values to *Candida albicans*. An increased understanding of the antifungal susceptibility profiles of *Candida* species isolated from CF patients may provide valuable knowledge about treatment of yeast infections in this patient group.

**IMPORTANCE:**

Our findings indicate that colonization with *Candida dubliniensis* and *Candida albicans* does not exert a significant impact on pulmonary function in individuals with cystic fibrosis. Furthermore, antifungal resistance among the isolated *Candida* species was generally low.

## INTRODUCTION

Cystic fibrosis (CF) is one of the most prevalent genetic disorders among Caucasians, affecting over 160,000 individuals worldwide (1). This condition arises from mutations in the cystic fibrosis transmembrane regulator (CFTR) gene located on chromosome 7. The CFTR gene encodes a large protein that regulates chloride transport across epithelial cell membranes in the respiratory and gastrointestinal tracts (2). CF primarily impacts cells that produce mucus, sweat, and digestive fluids. Anomalies in the CFTR protein lead to the secretion of a thick, sticky mucus in the respiratory tract, which facilitates the entrapment of bacterial and fungal cells. This creates an environment conducive to microbial colonization and subsequent infections by pathogenic bacteria and fungi. Respiratory infections are the leading cause of mortality and morbidity among CF patients.

Pathogenic bacteria such as *Pseudomonas aeruginosa*, *Staphylococcus aureus*, *Burkholderia* spp., *Achromobacter* spp., *Stenotrophomonas maltophilia,* and various fungi are typical opportunistic microorganisms recovered from the sputa of CF patients. Traditionally, great attention has been directed toward bacterial colonization and infection. However, in recent decades, fungi have increasingly been observed in CF patients, likely due to advanced and more standardized microbiological methods (3, 4). Prominent fungal colonizers in CF patients include *Candida albicans*, *Aspergillus fumigatus*, *Scedosporium* spp., and *Exophiala dermatitidis*, although the prevalence and diversity of fungi vary across countries and studies (4–6). *C. albicans* is one of the most common fungi causing healthcare-associated invasive fungal infections (7). Despite this, the clinical significance of yeasts in the respiratory tract of CF patients remains poorly understood, with limited publications addressing their impact. A recent study has associated the recovery of *C. dubliniensis* in CF patients with a deterioration in lung function (8).

Antifungal drug resistance in clinical settings presents an emerging global concern (9). Previously susceptible fungal species have now gained resistance to antifungal agents. There has also been a significant increase in the number of azole-resistant C. *glabrata* in patients with invasive fungal infections (10, 11). Moreover, the emergence of a multidrug-resistant fungus like *Candida auris* poses a substantial threat to human health partly attributed to extensive use of fungicides in agriculture (12). Antifungal susceptibility analysis is fundamental in initiating appropriate therapy and determining the susceptibility or resistance of fungal pathogens (13).

The presence and antifungal susceptibility profiles of *Candida* species in CF patients in the Nordic countries have not been thoroughly investigated. Understanding the epidemiology and clinical significance of pathogenic microorganisms in CF patients is crucial for diagnosis and treatment. The aim of this study was to investigate the prevalence of *C. dubliniensis* and *C. albicans* in sputum samples from CF patients. The association of *C. dubliniensis* with lung function measured by forced expiratory volume (FEV1) was also examined. Additionally, antifungal susceptibility of the two most commonly occurring *Candida* species, that is, *C. dubliniensis* and *C. albicans* to six antifungal agents—fluconazole, itraconazole, voriconazole, caspofungin, flucytosine, and amphotericin was assessed.

## MATERIALS AND METHODS

### Patients

Clinical data, including concomitant chronic colonization and lung function, were retrieved from the Swedish CF registry (Table 1). Data from each patient’s annual exam that was performed during the inclusion period were used. Lung-transplanted individuals were excluded from the analyses.

**TABLE 1:** Clinical features and demographic data of patients with cystic fibrosis included in this study

	All patients	Culture positive for *Candida dubliniensis*	Culture positive for *Candida albicans*	***P - value***
Patients, *n*	193	25	61	
Age, years, mean (SD)	29.7 (16.0)	27.4 (12.1)	29.2 (15.6)	*P* = 0.54
Male/female	113/80	12/13	34/27	*P* = 0.195
CFTR^*a*^ genotype				
F508del homozygote, *n* (%)	91 (47%)	10 (40%)	23 (38%)	*P* = 0.51
F508del heterozygote, *n* (%)	83 (43%)	13 (52%)	32 (52%)	*P* = 0.275
Other/other, *n* (%)	21 (10%)	2 (8%)	6 (10%)	*P* = 0.743
ppFEV1^*b*^, mean (SD)	78.5 (23.3)	73.6 (28.1)	73.3 (22.9)	*P* = 0.360
BMI^*c*^ kg/m^2^, mean (SD)	19.0 (3.1)	20.5 (3.1)	20.3 (3.5)	*P* = 0.666
Exocrine pancreas insufficiency	157 (81%)	24 (96%)	52 (85%)	*P* = 0.05
Patients with *P. aeruginosa*,*^d^ n* (%)	73 (39%)	15 (60%)	30 (49%)	*P* = 0.003
Patients with NTM,^*e*^ *n* (%)	11 (6%)	2 (8%)	2 (3%)	*P* = 0.601
CFTR modulator treatment				
All	84 (43%)	13 (52%)	23 (38%)	
Elexacaftor/tezacaftor/ivacaftor	15 (8%)	5 (20%)	6 (10%)	*P* = 0.037
Lumacaftor/ivacaftor	58 (30%)	5 (20%)	14 (23%)	*P* = 0.348
Tezacaftor/ivacaftor	3 (2%)	0 (0%)	1 (2%)	*P* = 1.0
Ivacaftor	3 (2%)	1 (0.5%)	0	*P* = 0.358
Other	5 (3%)	2 (8%)	2 (3%)	*P* = 0.091
Inhaled antibiotics	56 (29%)	11 (44%)	22 (35%)	*P* = 0.059
Antifungal therapy	8 (4%)	0 (0%)	0 (0%)	*P* = 0.569
No. of exacerbations in past year, mean (min–max)^*f*^	0.63 (0–5)	1.2 (0–3)	1.07 (0–5)	*P* = 0.76

^*a*^ CFTR, cystic fibrosis transmembrane conductance regulator.

^*b*^ ppFEV1, percent predicted forced expiratory volume in the first second.

^*c*^ BMI, body mass index.

^*d*^ *P. aeruginosa, Pseudomonas aerginosa* colonization both chronic and intermittent according to Leed’s criteria.

^*e*^ NTM, non-tuberculous Mycobacteria.

^*f*^ Exacerbations needing treatment with IV antibiotics in the last year, mean. Nine patients had both *C. albicans* and *C. dubliniensis*. *P*-value was adjusted for ppFEV1 using ANCOVA.

Routine examination of 193 CF patients (80 female and 113 male, 29.7 ± 16 years old) at the Department of Clinical Microbiology, Sahlgrenska University Hospital conducted between October 2021 to the end of October 2022, resulted in 968 culture-positive sputum specimens. None of the patients had received antifungal treatment prior to sampling. This study was conducted before the introduction of highly efficient modulator treatment, which may influence the prevalence of *Candida* in CF patients.

### Clinical specimens

All of the sputum samples were induced with the help of airway clearance breathing exercises, physiotherapy, or inhalation of bronchodilators and hypertonic saline solution. Fresh sputum samples were promptly processed at the routine diagnostic laboratory at the Department of Clinical Microbiology at Sahlgrenska University Hospital in Gothenburg, Sweden. This laboratory is accredited in accordance with the International Standard ISO 15189:2012 and ISO 22870:2016. The sputum samples were examined by light microscopy to evaluate cellular composition and assess sample quality. Gram staining was performed on sputum specimens. Samples containing a predominance of leukocytes were considered representative of lower respiratory tract secretions and included for further microbiological analysis.

The culturing methods concerning sputum samples from CF patients as well as the species identification methods used are accredited and meet the technical competence requirements outlined by the laboratory’s quality management system. The Swedish Board for Accreditation and Conformity Assessment (Swedac) conducts regular surveillance and performs a full reassessment every 4 years to ensure that the terms of accreditation are continually met (14).

### Isolation of bacteria and yeast from sputum samples

Sputum specimens were cultured as previously described (14). Briefly, sputum samples were liquefied by the addition of pancreatin (10 mg/mL in PBS) in a volume ratio of 1/1. The samples were then vortexed and incubated at room temperature for 15 min. The sample (100 µL) was inoculated on Horse blood agar, Streptococcus GBG agar, Blue Chrom agar staphylococcus, Sabouraud dextrose agar, Erythritol chloramphenicol (ECA) agar, Malt agar, and Haemophilus agar plates. The plates were incubated at 37°C and were examined for up to 2 days, except for Malt, ECA, and Sabouraud, which were incubated for 10, 20, and 4 days, respectively, for the detection of fungi.

### Identification of microorganisms

Fungal isolates were identified to the species level using matrix-assisted laser desorption ionization-time-of-flight mass spectrometry (VITEK-MS, bioMerieux, Marcy-l'Étoile, France) method according to the manufacturer’s instructions. Briefly, fungal colony biomass was collected from agar medium, using a sterile pointer and smeared onto a MS target spot; 1 µL of matrix solution (saturated cyano-4-hydroxycinnamic acid in water:acetonitrile:ethanol [1:1:1], acidified with 3% trifluoroacetic acid) was added to each biomass spot and allowed to crystalize. The reference strain *Escherichia coli* ATCC 8739 was included in the analysis as a quality control (15).

### Antifungal susceptibility testing

The antifungal susceptibility of *C. albicans* (*n* = 46) and *C. dubliniensis* (*n* = 15) was determined using Sensititre YeastOne panels (Trek Diagnostic System, Thermo Scientific, East Grinstead, West Sussex, UK). This method is based on the broth microdilution technique in 96-well microtiter plates. The plates contained twofold serial dilution of fluconazole (0.12–256 µg/mL), itraconazole (0.008–16 µg/mL), voriconazole (0.008–8 µg/mL), caspofungin (0.008–8 µg/mL), flucytosine (0.06–64 µg/mL), and amphotericin B (0.12–8 µg/mL). Antifungal susceptibility testing was performed according to the manufacturer’s instructions. Briefly, 100 µL of *Candida* inoculum in buffer was added to each well. The microplates were covered and incubated at 37°C for 24 h. The minimum inhibitory concentration (MIC) was determined as the lowest concentration of antifungal agents at which the color in the microtiter well changed from red to blue. *Candida krusei* ATCC 6258 was included as a control organism in all experiments.

### Statistical analysis

Fisher’s exact test, independent sample *t*-test and Kruskal-Wallis test were performed using GraphPad Prism (GraphPad Software, San Diego, CA). ANCOVA analysis were used to adjust covariates. A *P* value of 0.05 was considered to be statistically significant in univariate analyses.

## RESULTS

### Isolation of fungi from sputum specimens

**Fig 1:**
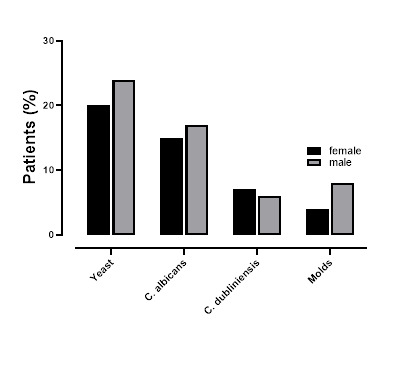
Isolation of yeast (including *Candida* subsp., molds, *Candida albicans*, and *Candida dubliniensis* from sputum samples in patients (male [*n* = 113] and female [*n* = 80]) with cystic fibrosis. No statistically significant difference was found in fungal colonization between men and women.

All samples were cultured and analyzed according to established clinical routine. A total of 193 CF patients participated in the study, with 37 patients providing a single sputum sample, 61 patients contributing 2–4 samples, and 95 patients submitting 5–15 samples each (Table 2). The fungal isolates from the sputum of CF patients are detailed in Table 3. Yeasts were identified in 44% of the patients, with *C. dubliniensis* (13%) and *C. albicans* (32%) being the most prevalent species. Additionally, *E. dermatitidis* was isolated from 12% of the patients. Bacterial colonization was also observed, with *S. aureus* (61%), *P. aeruginosa* (27%), and mucoid *P. aeruginosa* (14%) being the most common (Table 3). No significant difference in fungal colonization was observed between male and female patients (Fig. 1).

**TABLE 2:** Number of sputum specimens isolated from 193 patients with cystic fibrosis during the period between 1 October 2021 and 31 October 2022

Number of patients	Number of analyzed sputum specimens
37	1
21	2
20	3
20	4
17	5
16	6
13	7
21	8
7	9
5	10
7	11
3	12
3	13
2	14
1	15

**TABLE 3:** Bacteria and fungi isolated from sputum specimens (*n* = 968) from 193 patients with cystic fibrosis

Microorganisms	Patients *n* (%)	Specimens *n* (%)
Yeast (incl. *Candida* spp.)	85 (44)	209 (21.6)
*Candida albicans*	61 (31.6)	143 (14.8)
*Candida dubliniensis*	25 (12.9)	45 (4.7)
Molds	23 (11.9)	76 (7.9)
*Exophiala dermatitidis*	24 (12.4)	111 (11)
*Staphylococcus aureus*	118 (61)	418 (43.2)
*Pseudomonas aeruginosa*	52 (26.9)	207 (21.4)
*Pseudomonas aeruginosa mucoid*	32 (16.6)	128 (13.2)
*Haemophilus influenza*	26 (13.5)	44 (4.6)
*Stenotrophomonas maltophilia*	12 (6.2)	28 (2.9)
*Burkholderia* spp.	7 (3.6)	23 (2.4)

**Fig 2:**
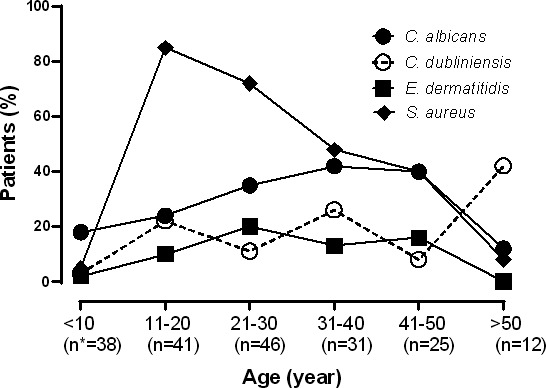
Age distribution of patients with *C. dubliniensis*, *C. albicans*, *E. dermatitidis,* and *S. aureus* in samples collected from airway of patients with cystic fibrosis. *Number of patients in each age group.

The age distribution of patients colonized by *C. dubliniensis*, *C. albicans*, and *S. aureus* (the most common bacterium isolated from CF patients) is illustrated in Fig. 2. Colonization by *C. dubliniensis* increased steadily with age but showed a significant rise in patients over 50 years of age. In contrast, *C. albicans* colonization peaked in patients aged 31–40 years. *S. aureus* was most frequently isolated from younger patients, particularly those aged 11–20 years.

### Antifungal susceptibility profiles

The susceptibility of *C. dubliniensis* and *C. albicans* to antifungal agents was analyzed, with MIC_50_ (the concentration of an antimicrobial agent at which 50% of fungal isolates are inhibited) and MIC_90_ (the concentration of an antimicrobial agent at which 90% of fungal isolates are inhibited) values presented in Table 4. Voriconazole exhibited the lowest MIC values against both *C. dubliniensis* and *C. albicans*. The MIC_90_ values for fluconazole, itraconazole, and voriconazole were slightly higher (by one dilution) for *C. dubliniensis* compared to *C. albicans* (Table 4). Applying the EUCAST clinical breakpoint to the MIC results, we found that 5 out of 45 of *C. albicans* (11%) and 3 out of 15 *C*. *dubliniensis* isolates (20%) were resistant to itraconazole.

**TABLE 4:** The concentration of antifungal agents required to inhibit the 50% and 90% of *Candida* isolates (MIC_50_ and MIC_90_) and EUCAST clinical breakpoints for *C. albicans* and *C. dubliniensis*

Antifungal agents	*Candida albicans*				*Candida dubliniensis*			
	MIC_50_^*a*^	MIC_90_^*b*^	EUCAST breakpoints		MIC_50_	MIC_90_	EUCAST breakpoints	
			S<	R>			S<	R>
Amphotericin	0.5	0.5	1	1	0.25	0.5	1 1	1
5-Flucytosine	0.06	0.25	ND^*c*^		0.06	0.06	ND	
Fluconazole	0.25	0.5	2	4	0.5	1	2	4
Itraconazole	0.06	0.06	0.6	0.6	0.06	0.120	0.06	0.06
Voriconazole	0.008	0.008	0.06	0.25	0.008	0.015	0.06 0.25	0.25
Caspofungin	0.120	0.120	ND		0.25	0.25	ND	

^*a*^ MIC_50_, the concentration of antifungal agents necessary to inhibit 50% of fungal isolates.

^*b*^ MIC_90_, the concentration of antifungal agents necessary to inhibit 90% of fungal isolates.

^*c*^ ND, not determined.

### Clinical data and lung function

Among the CF patients with at least one positive culture for *C. albicans*, the mean FEV1 was 73.3% of predicted value (standard deviation = 23) (Table 1). For those with at least one positive culture of *C. dubliniensis,* the mean FEV1 was 73.6% of predicted value (standard deviation = 28). There were no significant differences in lung function compared to patients without a positive culture for *C. albicans or C. dubliniensis* or compared with those without *Candida*. Additionally, no difference in lung function was observed between patients with a single positive culture for *C. dubliniensis* and those with multiple positive cultures during the study period (*P* = 0.15, data not shown). In those with positive cultures for *C. dubliniensis*, concomitant chronic colonization with *P. aeruginosa* was found in 15 patients (60%), *S. aureus* in 14 (56%), with 9 patients being colonized by both bacteria. Two patients (8%) were colonized with mycobacteria. No differences were found regarding the use of antifungal agents, inhaled antibiotics between the groups during the study period. The number of exacerbations needing IV antibiotics was slightly higher in patients with *C. dubliniensis,* but the difference was not significant after adjustment for lung function. Exocrine pancreas insufficiency and the use of highly effective CFTR modulator therapy with elexacaftor/tezacaftor/ivacaftor was more common in those with *C. dubliniensis*.

## DISCUSSION

In our study, *C. dubliniensis* was the second most prevalent *Candida* species recovered from CF patients with a frequency of 13%. This is slightly higher than the 11% reported by Al Shakirchi et al., who also found that the persistence of *C. dubliniensis* in the respiratory tract was associated with deteriorated lung function over 3 years of follow-up (8). In the present cross-sectional study, neither the presence of *C. albicans* nor *C. dubliniensis* in sputum was associated with a lower lung function. The use of antifungal treatment, intravenous or inhaled antibiotics was not higher in those with *Candida species* in sputum; however, we did note that the use of highly effective CFTR modulator therapy with elexacaftor/tezacaftor/ivacaftor was more common in those with *C. dubliniensis*. During the time of data collection, the use of highly effective CFTR modulator therapy was not widespread in Sweden and only those with very severe disease had access to treatment. This treatment typically improves lung function and may explain why we did not find any difference in lung function indices associated with *C. dubliniensis*.

*C. albicans* is a common member of the human commensal flora in gut and mucosal surfaces (16–18). In our study, 32% of patients were culture positive for *C. albicans*. In a multicenter study by Schwarz et al., a higher prevalence of *C. albicans* in CF patients has been reported from Austria, France, and Italy (5). It is important to note that a positive culture for *Candida* in these patients does not necessarily indicate an active infection but may instead reflect colonization.

The prevalence of *E. dermatitidis* in CF patients was lower (11%) in this study compared to our previous results (19%) (4). Variations in the demographic characteristics or health status of the study participants can impact the prevalence rates. Additionally, external environmental conditions, such as climate and geographical location, can influence the prevalence of *E. dermatitidis*. Studies conducted in different environmental settings may report varying prevalence rates due to differences in environmental exposure to the fungus (19).

The antifungal susceptibility testing demonstrated that voriconazole showed the lowest MIC against *C. dubliniensis* and *C. albicans*. We found that 11% of *C. albicans* and 20% of *C. dubliniensis* were classified as resistant when EUCAST breakpoints were applied. The MIC values obtained for *Candida* species to itraconazole were only one to two dilutions higher than the defined breakpoint by EUCAST (0.06 µg/mL for itraconazole). However, the *Candida* species were classified as susceptible when CLSI clinical breakpoints for itraconazole were used. Discrepancies between EUCAST and CLSI standards methods, such as differences in glucose concentration in growth medium, and fungal cell density in microplates, as well as the use of a spectrophotometer in the EUCAST method may contribute to these variations. However, earlier studies have reported that MIC values obtained by the Sensititre method were highly comparable to the CLSI reference method (20).

Respiratory tract infections significantly contribute to mortality and morbidity in CF patients. Yeasts, especially *C. albicans* and *C. dubliniensis*, are common colonizers of CF airways. Despite the presence of *C. dubliniensis*, lung function data indicated no significant difference in FEV1 between patients with and without *C. dubliniensis* colonization, suggesting it may not directly impact lung function. Moreover, the resistance to antifungal agents was found to be low in our study, except for itraconazole (when EUCAST clinical breakpoints were applied). Further research with more clinical isolates is needed to better understand antifungal susceptibility profiles, which would enhance treatment strategies and improve outcomes for CF-associated fungal infections. This study is limited by the low number of *Candida* species in the antifungal susceptibility testing. Another limitation is the cross-sectional nature of the clinical data from the patient registry, which prevents a more detailed analysis of change over time and indeed if any variability in lung function during the year could be connected with findings in the sputum. Further studies involving a larger number of clinical isolates are warranted to understand the antifungal susceptibility profiles of *Candida* species isolated from CF patients. This would provide valuable knowledge about treatment and improve outcomes in fungal infections in CF.
